# Cytotoxic effect of potential probiotic *Lactiplantibacillus plantarum* KUMS-Y8 isolated from traditional dairy samples on the KB and OSCC human cancer cell lines

**DOI:** 10.1016/j.heliyon.2023.e20147

**Published:** 2023-09-15

**Authors:** Babak Haghshenas, Yousef Nami, Amir Kiani, Nesa Moazami, Omid Tavallaei

**Affiliations:** a Regenerative Medicine Research Center (RMRC), Health Technology Institute, Kermanshah University of Medical Sciences, Kermanshah, Iran; b Department of Food Biotechnology, Branch for Northwest and West Region, Agricultural Biotechnology Research Institute of Iran, Agricultural Research, Education and Extension Organization (AREEO), Tabriz, Iran; c Students Research Committee, Faculty of Pharmacy, Kermanshah University of Medical Sciences, Kermanshah, Iran; d Pharmaceutical Sciences Research Center, Health Institute, Kermanshah University of Medical Sciences, Kermanshah, Iran

**Keywords:** Anticancer, Apoptosis, Oral cancer, *Lactobacillus*, Probiotics

## Abstract

Oral cancer is one of the leading causes of death worldwide, and its prevalence is especially high in developing countries. As an oral cancer treatment, traditional therapies are commonly used. Nonetheless, these treatments frequently result in a variety of side effects. As a consequence, there is an urgent need to enhance oral cancer therapies. Probiotics have recently demonstrated intriguing properties as therapeutic options for cancer treatment. Thus, the purpose of this study was to investigate the anticancer effect of probiotic *Lactobacillus* strains on the mouth epidermal carcinoma cells (KB) and oral squamous cell carcinoma (OSCC) cell lines. In this study, we looked at 21 *Lactobacillus* strains isolated from traditional dairy products in the Kermanshah province of western Iran to see if they had any inhibitory effects on oral cancer cell lines in vitro. We isolated and characterized *Lactobacillus* strains before assessing and comparing their probiotic potential and safety. Using the MTT assay, the bacterial extract was then prepared and used as an anti-proliferative agent on oral cancer (KB and OSCC) and normal (fibroblast and human umbilical vein endothelial cells (HUVEK) cell lines. Finally, acridine orange/ethidium bromide staining was used to determine whether cell death was caused by apoptosis. Four *Lactobacillus* isolates (C14, M22, M42, and Y8) were shown to have beneficial probiotic qualities. *Lactobacillus* extracts (of a protein nature) decreased the survival and proliferation of the KB and OSCC cancer cell lines (dose- and time-dependent) by inducing apoptosis, with no basic damaging effects on normal cells. The staining with acridine orange/ethidium bromide revealed that the cell death was caused by apoptosis. Furthermore, of the four *Lactobacillus* strains examined, isolate Y8 (*Lactiplantibacillus plantarum*) showed the strongest probiotic potential for suppressing KB and OSCC cell proliferation when compared to anticancer medicines (doxorubicin and paclitaxel). The current research found that *Lactobacillus* extract might reduce the growth and viability of the KB and OSCC cancer cell lines by inducing apoptosis, increasing the survival rate of oral cancer patients.

## 1 Introduction

Globocan 2020 says that oral cancer is a common type of cancer, with 177,757 deaths and 377,713 new cases each year. Oral cancer is quite common in South-Central Asia [1]. Oral squamous cell carcinoma (OSCC) is the most frequent oral cancer, accounting for more than 90% of all oral malignancies in terms of clinical indications and interest [2]. Infections, inflammation, HPV, radiation, alcohol, smoking, immunosuppression, nutrition, and heredity are all important risk factors for oral cancer. Even though there are new ways to treat oral cancer, the 5-year survival rate remains below 50% in most countries. Alcohol and tobacco use are two of the most important risk factors for oral cancer [3]. Several inflammatory pathways, such as cyclooxygenase (COX)-2, Janus kinase/signal transducer nuclear factor-B (NF–B), mitogen-activated protein kinases (MAPK), and activators of transcription (JAK/STAT), may also be involved in the development of oral cancer. Patients with oral cancer have significantly higher levels of Candida albicans genotype A strains than non-cancer cases, and these strains have also been linked to leukoplakia lesions. In addition, immunosuppression has been linked to the spread of oral cancer in patients undergoing bone marrow transplantation and renal transplantation. In spite of progress in therapeutic methods for oral cancer, the survival frequency has not increased remarkably over the last few decades. So, we need new and effective ways to treat cancer to stop it from spreading [4].

Probiotics, being living microbes, may impact the health of their hosts when appropriate quantities are swallowed [5]. The main criteria for choosing probiotics are their acid and bile salt tolerance, safety, capacity to adhere to and colonize the digestive tract, and host health benefits [6,7]. Goldin and Gorbach were the first to demonstrate a link between a lower incidence of colon cancer and a *Lactobacillus*-enriched diet [8]. Probiotics have gotten a lot of attention because they can slow down the growth and death of cancer cells both in vivo and in vitro. Future applications of these qualities in novel therapies may serve as a replacement for more intrusive treatments such as radiation or chemotherapy [9]. Lactobacilli have a variety of functions, including reducing the activity of tumor-causing enzymes, increasing the host's immune system, and producing metabolites that aid in host and pathogen resistance [10]. So yet, no study has been conducted to determine the impact of *Lactobacillus* bacteria on oral malignancies in people. We tried to figure out how well Lactobacillus bacteria fight cancer by using KB and OSCC oral cell lines as an in vitro model system.

## 2 Material and method

### 2.1 *Lactobacillus* strains isolation and culture

Bacterial strains were found in 150 samples of raw milk, yogurt, and typical cheese. Each of the 50 samples was obtained randomly from various locations around Kermanshah province. The samples were transported to the lab and kept at 4 °C. For a better and more efficient separation of bacteria from solid particles, samples (10 g) were suspended in a sterile trisodium citrate solution (90 mL). After 1 h, 5 mL of the aforementioned solution was added to 100 mL of de Man Rogosa & Sharpe (MRS) culture medium to enrich and enhance the original bacterial population. Bacterial strains were propagated by anaerobic growth in MRS medium for 24 h at 37 °C and cultivated in MRS agar medium under the same conditions as described before. Following that, bacterial colonies were subjected to preliminary biochemical and morphological assays such as cell morphological analysis, catalase testing, and Gram staining.

### 2.2 Acid and bile salt tolerance test

Ten mL of each 24-h bacterial culture in MRS medium was centrifuged at 4000×*g* for 5 min to assess bacterial resistance to low pH, oral pH, and bile salts. After discarding the supernatant, cell pellets were gently stirred for 3 h in 10 mL of low pH solution (pH 2.5 at 37 °C) and oral pH solution (pH 3.6 at 37 °C), then for 4 h in 10 mL of high bile salt concentration solution (pH 8.6 at 37 °C with oxgall 3.0% w/v). To reduce the number of bacterial strains investigated, the first selection was conducted by analyzing optical densities using the Yang et al. (2017) approach. The optical density of control and treated strains was measured at 600 nm in a spectrophotometer (Eppendorf, Germany), and tolerance to acidic conditions and bile salt was estimated by referring to the percentage of bacterial survival using equation (1):

(1) Survival percentage = [OD after treatment / OD before treatment] × 100

Then, the samples of bacteria with the best initial results were chosen to be tested under strict digestion conditions. Pepsin at a final concentration of 5% (w/v) was added to chosen strains with an initial cell population of 1.2–6.3 × 10^9^ CFU/mL (pH 2.5 at 37 °C), and the cells were incubated at 450×*g* for 2 h to measure survival in gastric conditions. To simulate intestinal digesting conditions, a solution of bile salts and pancreatin in concentrations of 0.3 and 0.1% (w/v) (pH 6.0 at 37 °C) was also added and incubated for 3 h with moderate stirring at 450 g. The samples were diluted and cultured in three replicates on MRS agar medium for 48 h at 37 °C before counting bacterial clones. equation (2) was used to determine the survival rate of bacterial strains under gastrointestinal conditions:

(2) Survival percentage = (log CFU N_1_ / log CFU N_0_) × 100

Where N_1_ represents the total number of bacterial clones following therapy for gastrointestinal diseases and N_0_ represents the total number of bacterial clones before to treatment.

### 2.3 Inhibitory effects of *Lactobacillus* bacteria against pathogens

The well diffusion method was used to test the activity of isolated potential probiotic strains against common human pathogens like *Yersinia enterocolitica* (ATCC 23715), *Escherichia coli* (PTCC 1276), *Streptococcus mutans* (PTCC 1683), *Bacillus subtilis* (ATCC 19652), *Staphylococcus aureus* (ATCC 25923), *Klebsiella pneumonia* (PTCC 1053), and *Listeria monocytogenes* (PTCC 1234). In this procedure, half McFarland (1.5 × 10^8^ CFU/mL) concentrations of each pathogen were cultivated on Müller-Hinton agar medium, and then wells were made on the inoculation medium. Each putative probiotic strain's wells were filled with 100 μL of filtered cell-free extract (supernatant) from an overnight culture and then incubated at 37 °C overnight. Finally, the pathogen inhibition halo was measured using a digital caliper. Nami et al. [11,12] modified their approach to assess the type of anti-pathogenic chemicals in extracts of each possible probiotic strain. Cell-free extracts of putative probiotic strains were prepared by centrifugation for 20 min at 4000 g at 4 °C. After correcting the pH to 6.2, the extracts were treated for 2 h at 37 °C with 1 mg/mL proteinase K and catalase before being tested for anti-pathogenic activity using the agar-well diffusion technique.

### 2.4 Evaluation of sensitivity to antibiotics

To determine the antibiotic sensitivity of potential probiotic strains, the method of diffusion in agar was used on several high-dose and clinically essential antibiotics containing Cefixime (5 μg), Amoxicillin (25 μg), Azithromycin (15 μg), Doxycycline (30 μg), Trimethoprim sulfamethoxazole (75 μg), Ciprofloxacin (5 μg), Cephalexin (30 μg), Amoxicillin-clavulanic acid (10 μg), and vancomycin (30 μg). The diameter of the inhibitory halo around the discs was measured using a digital caliper after culturing potential probiotic strains overnight in agar MRS medium (37 °C) and placing antibiotic discs on inoculated media.

### 2.5 Investigation of cell surface hydrophobicity

The Nami et al. approach was used to assess the hydrophobicity of the cell surface [13]. Bacterial strains were centrifuged overnight at 6000×*g* for 10 min in this procedure, and 1 × 10^8^ CFU/mL of precipitated bacteria were suspended in 3 mL of phosphate buffer solution (PBS). At 600 nm, the first adsorption was detected (A_0_). The bacterial suspension was then treated with 1 mL of xylene (Merck, Germany), which was vortexed for 2 min. The phases were separated for 1 h at 37 °C, and aqueous phase adsorption was measured (A_1_). In three replications of this experiment, the hydrophobicity of the cell surface was calculated as a percentage using equation (3):

(3) Hydrophobicity (%) = (1 - A_1_/A_0_) 100.

### 2.6 *In vitro* cell adhesion assay

The Caco2 cell line was used to test the strains' ability to stick to human epithelial cells. Caco2 cells were grown under controlled conditions at 37 °C in 1640-RPMI (Sigma) medium with 10% heat-inactivated bovine fetal serum and 50 units/mL penicillin-streptomycin. Caco2 cells were cultured on glass sheets in 6-well tissue culture plates to measure bacterial germination, and after 24 h of incubation at 37 °C (5% CO_2_), the monolayers were washed twice with sterile PBS (pH 7.4) and 10 mL of bacterial suspension (1 × 10^7^ CFU/mL) was added to each plate. Inoculated cell plates were incubated at 37 °C for 2 h before being washed three times with PBS buffer (pH 7.4) to remove non-adherent bacteria. Adherent bacteria were isolated and resuspended in 10 mL of saline solution using a trypsin-EDTA solution (0.05%). The bacteria were then grown in repeated dilutions on MRS agar for 24–48 h at 37 °C. To figure out the percentage of adhesion, the number of bacterial cells that stuck to the surface was compared to the total number of bacterial cells. The cell adhesion test was done three times, and the mean and standard deviation (SD) of the results were given.

### 2.7 Investigation of cholesterol absorption

The potential of probiotic strains to lower total cholesterol was evaluated using the *o*-phthaldehyde technique published by Miremadi et al. [14]. The strains were incubated for 20 h at 37 °C in MRS culture media with 150 g/mL water-soluble cholesterol (polyoxyethylcholesteryl sebacate; Sigma) and 0.3% bile salt (oxgall bile). The bacterial cells were then centrifuged for 15 min at 4300×*g*, and the residual cholesterol in the aqueous phase was quantified using the *o*-phthaldehyde technique.

### 2.8 Investigation of hemolytic activity

The hemolytic activity of possible probiotic strains was measured with the Abedi et al. method [15]. To measure hemolytic activity, three categories were used: light halos around the colony for β-hemolysis, a bright halo around the colony for α-hemolysis, and no halo around the colony for γ-hemolysis.

### 2.9 The autoaggregation and coaggregation ability evaluation

The ability of autoaggregation of strains was measured by Angmo et al. (Angmo, Kumari, & Bhalla, 2016). To determine the percentage of autoaggregation, equation (4) was used. In the above equations, A_0_ represents adsorption at time 0, and A_t_ represents adsorption at time t. Also, coaggregation was determined by Zuo et al.'s method with equation (5) [16]:

(4) % = 1 − (A_t_/A_0_) × 100

(5) % = (A_0_ – A_t_) / A_t_ × 100 [16].

### 2.10 Cell culture conditions and treatments

The Pastor Institute (Iran) gave KB and OSCC oral cancer cells, as well as normal fibroblast and HUVEK cells. KB and fibroblast cells (PAN-Biotech, Cat. No. P06-07100) were grown in Eagle's Minimum Essential Medium (EMEM) (Sigma-Aldrich, Cat. No. M4655) with 10% fetal bovine serum (FBS) (Gibco, Cat. No. 10270-106) and 1% penicillin-streptomycin solution added. Cells were incubated in 5% CO_2_ at 37 °C. The cells were detached with trypsin/EDTA 0.25% when they reached 80% confluence (PAN-Biotech, Cat. No.: P10-029100). For 24 h, the cells were grown in 96-well plates (2000 cells per well). The EMEM medium was then withdrawn and replaced with 200 L of varying concentrations of possible probiotic suspension (without antibiotic solution) or incubated consecutively for 24, 48, and 72 h. The control groups included cancer and normal cells that had not been treated with probiotics or had been treated with MRS, anticancer medicines (doxorubicin and paclitaxel), and an in-market probiotic strain (*Lactobacillus acidophilus* PTCC 1643).

### 2.11 Assessment of cell proliferation using MTT assay

Cell proliferation was measured using the 3-(4,5-dimethylthiazol-z-yl)-2,5-diphenyltetrazolium bromide (MTT; Sigma-Aldrich, Cat. No. M5655-1G) assay. In total, 180 μL of cells (2 × 10^4^ cells/mL) were planted onto 96-well plates and incubated for 24 h. The cells and supernatants were then added in varied quantities (20 μL) and incubated at 37 °C. MTT solution (5 mg/mL, 20 μL) was added to each well after 24, 48, and 72 h of incubation and incubated for an additional 3 h. After discarding the medium, the formazan crystals were resolved in 150 μL of dimethyl sulfoxide (Sigma-Aldrich, Cat. No: 472301-1L). MTT was changed to formazan using metabolically live cells, and its absorbance was measured at 570–630 nm using an ELISA reader (Bioteck, Synergy H1, USA). This test was carried out over 24 h, 48 h, and 72 h with varied concentrations (1–25 g/mL) of extracted metabolites and six replicates in each plate. In addition, the absorbance values of untreated, MRS-treated, treated with an under-market probiotic strain (*Lactobacillus acidophilus* PTCC 1643), and drug-treated (doxorubicin and paclitaxel) cells as negative and positive controls were measured under the same conditions.

The MTT test was used to determine and characterize anti-cancer metabolites (active proteins) in oral cancer (KB and OSCC) and normal (fibroblast and HUVEK) cell lines after 48 and 72 h of incubation with six repetitions. The supernatants were treated with pronase (Roche Applied Science) at a dosage of about 1 mg/mL. They were incubated in a water bath or incubator for 30 min at 37 °C. The affinity of active proteins was measured after full protein digestion by comparing absorbance values for normally treated, pronase-treated, and untreated cells with drug-treated cell lines. equation (6) was used to compute the cell viability percentage for each sample:

(6) Percentage of cell viability = (sample-blank) / (control-blank) × 100%

### 2.12 Dual acridine orange/ethidium bromide (AO/EB) fluorescent staining

To stain with AO/EB, cells were cultured in 24-well plates and treated with the extracts' IC50 concentration. After 12 h, the cells were harvested and washed three times with cold PBS. They were suspended in a 100-L mixture of acridine orange and ethidium bromide (1:100 mg/mL). Finally, 10 μL of stained cell suspension was placed on a slide and healthy and apoptotic cells were identified using a fluorescent microscope (Bioteck, CellCytaion, USA).

### 2.13 Molecular identification and DNA sequencing

Amplification of the 16S rRNA gene (1500 bp) of the strains was performed using a pair of primers (Hal6F/Hal6R) (F: 5′-AGAGTTTGATCMTGGCTCAG-3′ and R: 5′-TACCTTGTTAGGACTTCACC-3′) previously described by Nami et al. [17]. The PCR program was performed as follows: initial denaturation at 95 °C for 5 min, followed by 30 cycles of denaturation at 94 °C for 60 s, primer annealing at 57 °C for 60 s, extension at 72 °C for 120 s, and a final extension at 72 °C for 10 min. The PCR products were electrophoresed on a 1% agarose gel and stained with ethidium bromide. The PCR products were sent to the Macrogene DNA Sequencing Service (Korea) for sequencing. The sequences obtained after analysis of the amplified PCR products were aligned with Clustal W and then compared to the strains retrieved from the GenBank databases.

### 2.14 Statistical analysis

All of the investigations were repeated three times using a totally random factorial design, and the findings were reported as the mean and standard deviation. SPSS 18 was used to look at the data and figure out how important the samples were. Duncan's multiple range test was used to examine how the means changed when variables had a significant influence (P < 0.05).

## 3 Results

### 3.1 Biochemical and morphological analysis of bacteria

Bacterial colonies with a white to creamy hemispherical shape were isolated. From the current colonies, 21 g-positive and catalase-negative *Bacillus* species were isolated and grown as Lactobacilli in MRS medium under anaerobic conditions. These 21 isolates were chosen and conquered for further investigation (Table 1).

**Table 1:** Dairy origin, catalase test, gram staining, and survival rate of isolated *Lactobacillus* strains after 3–4 h of incubation at 0.3% bile salt, and pH 2.5–6.6

Survival rates = ([OD_600_ (3–4 h)/OD_600_ (0 h)] × 100)						
Isolated strains	Origin	Catalase test	Gram staining	Tolerance after 4 h at 0.3% bile	Tolerance after 3 h at pH 2.5	Tolerance after 3 h at pH 6.6
C3	Cheese/Sheep	Negative	*Gram*-positive	57.12 ± 1.43^g^	40.22 ± 1.38^h^	111.88 ± 1.18^ef^
C12	Cheese/Sheep	Negative	*Gram*-positive	31.27 ± 0.97^j^	29.18 ± 1.29^jk^	113.97 ± 0.75^e^
C14	Cheese/Sheep	Negative	*Gram*-positive	*106.80 ± 1.44a*	*90.36 ± 1.01a*	120.31 ± 4.24^b^
C29	Cheese/Sheep	Negative	*Gram*-positive	*87.15 ± 2.08c*	*78.05 ± 2.38c*	102.53 ± 1.14^ijk^
C32	Cheese/Sheep	Negative	*Gram*-positive	12.24 ± 2.19^m^	18.73 ± 0.98^n^	107.78 ± 1.28^g^
C35	Cheese/Sheep	Negative	*Gram*-positive	*33.84 ± 1.59ij*	*27.85 ± 4.32k*	98.62 ± 0.94^l^
C42	Cheese/Sheep	Negative	*Gram*-positive	*31.98 ± 1.18j*	*23.86 ± 1.56l*	103.76 ± 1.56^hij^
M20	Milk/Sheep	Negative	*Gram*-positive	57.86 ± 3.24^g^	*46.44 ± 1.36g*	113.13 ± 0.77^e^
M22	Milk/Sheep	Negative	*Gram*-positive	*93.31 ± 1.25b*	*80.78 ± 1.19c*	110.38 ± 1.38^f^
M35	Milk/Sheep	Negative	*Gram*-positive	62.18 ± 2.07^f^	51.19 ± 1.39^f^	118.87 ± 1.87^c^
M37	Milk/Sheep	Negative	*Gram*-positive	*16.28 ± 0.74l*	*9.08 ± 0.60op*	101.12 ± 2.16^ijkl^
M42	Milk/Sheep	Negative	*Gram*-positive	*95.63 ± 1.23b*	*84.58 ± 0.65b*	117.34 ± 1.82^cd^
M48	Milk/Sheep	Negative	*Gram*-positive	39.09 ± 0.76^i^	30.45 ± 1.44^j^	95.76 ± 2.09^m^
Y4	Yogurt/Sheep	Negative	*Gram*-positive	21.27 ± 1.29^k^	21.09 ± 0.49^m^	105.58 ± 1.44^h^
Y8	Yogurt/Sheep	Negative	*Gram*-positive	*77.41 ± 0.89d*	*71.78 ± 1.24d*	125.29 ± 1.91^a^
Y10	Yogurt/Sheep	Negative	*Gram*-positive	15.92 ± 0.54^l^	11.83 ± 1.78^o^	103.26 ± 1.12^ij^
Y40	Yogurt/Sheep	Negative	*Gram*-positive	*70.55 ± 1.64e*	*60.73 ± 2.42e*	120.79 ± 2.23^b^
Y41	Yogurt/Sheep	Negative	*Gram*-positive	58.89 ± 1.23^g^	45.27 ± 1.09^g^	115.35 ± 1.04^d^
Y43	Yogurt/Sheep	Negative	*Gram*-positive	40.13 ± 0.29^i^	28.13 ± 2.46^k^	100.96 ± 2.28^jkl^
Y48	Yogurt/Sheep	Negative	*Gram*-positive	*44.73 ± 0.59h*	*35.79 ± 0.76i*	99.13 ± 1.78l^l^
Y50	Yogurt/Sheep	Negative	*Gram*-positive	*20.24 ± 1.61k*	*7.90 ± 1.46p*	103.94 ± 1.51^hi^

*Values are mean ± standard error of triplicates. ^a-p^ Means in the same column with different lowercase letters differed significantly (P < 0.05).

### 3.2 The tolerance of lab isolates against acid, bile salt and digestive conditions

The vitality of probiotics in the colon and stomach is a key characteristic. The bile salt and acidic environments exhibited varying effects on the proliferation of all 21 isolates (Table 1). Finally, four isolates were chosen for further research. Table 2 displays the viability of selected isolates in gastric and intestinal conditions. The findings revealed that the chosen strains had high longevity in the early hours of incubation. A slight decrease in the amount of logarithmic CFU/mL was observed after 1 h under gastric conditions, but a further decrease was observed between 1 and 2 h. Furthermore, at the start of the in vitro digestion, the average counts ranged from 9.123 ± 0.14 to 9.802 ± 0.16 Log CFU/mL. The isolates C14, M22, M42, and Y8 had viable numbers of 5.749 ± 0.12, 4.474 ± 0.17, 4.803 ± 0.15, and 3.137 ± 0.11 Log CFU/mL at the end of the gastric condition, respectively. Under intestinal conditions, all four isolates showed good viability in the first hour, but there was a decrease in log CFU/mL between 2 and 3 h. The isolates C14, M22, M42, and Y8 had a viable number of 6.310 ± 0.16, 5.295 ± 0.21, 5.078 ± 0.13, and 3.953 ± 0.18 Log CFU/mL at the end of the intestinal condition, respectively.

**Table 2:** Re-screening and survival rate (%) of isolated *Lactobacillus* strains under gastric and intestinal digestive conditions.

Isolated strains	Final counts (log CFU/mL) after incubation in gastric conditions				Final counts (log CFU/mL) after incubation at intestinal conditions				
	0 h	1h	2h	SR (%)	0 h	1h	2h	3 h	SR (%)
**C14**	9.273 ± 0.18	8.773 ± 0.15	5.749 ± 0.12	62^a^	9.418 ± 0.21	9.124 ± 0.19	6.618 ± 0.14	6.310 ± 0.16	67^a^
**M22**	9.123 ± 0.14	8.429 ± 0.11	4.474 ± 0.17	52^b^	9.129 ± 0.17	8.718 ± 0.16	5.723 ± 0.13	5.295 ± 0.21	58^b^
**M42**	9.418 ± 0.12	8.711 ± 0.19	4.803 ± 0.15	51^b^	9.068 ± 0.18	8.626 ± 0.18	5.418 ± 0.11	5.078 ± 0.13	56^b^
**Y8**	9.802 ± 0.16	8.724 ± 0.13	3.137 ± 0.11	32^c^	9.413 ± 0.19	8.621 ± 0.19	4.732 ± 0.14	3.953 ± 0.18	42^c^

*Values followed by the same letters are not significantly different (P < 0.05). Statistical analysis of each formulation was done separately. SR: Survival Rate.

### 3.3 Antibacterial activity

Table 3 shows the antagonistic activity of four isolated strains against eleven pathogens at varied pH (neutral and natural pH) and conditions (catalase and proteinase K treatment). The M22, M42, and Y8 strains were shown to have considerable anti-pathogenic action and to suppress the development of all pathogens (Table 3). C14, on the other hand, had excellent antagonistic action and prevented the development of 10 pathogens except S. salivarius. The C14 and M42 strains showed no anti-pathogenic activity against *S. sanguinis*, *S. sobrinus*, or *S. mutans* after changing the pH to 6.8. Furthermore, strain M22 was unable to inhibit the growth of *B. subtilis*, and strain Y8 was unable to inhibit the growth of *S. salivarius* and *B. subtilis*. Because of this, acid production may be the way these strains stop the spread of the diseases mentioned above.

**Table 3:** The inhibitory effect of isolated *Lactobacillus* strains against pathogens.

Pathogens	Diameter of inhibition zone (mm)			
	C14	M22	M42	Y8
***S. sanguinis***	12.0 ± 0.3^b^	12.1 ± 0.3^b^	16.3 ± 0.3^a^	12.0 ± 0.4^b^
***S. salivarius***	0.0 ± 0.0^c^	9.0 ± 0.2^b^	12.0 ± 0.3^a^	12.3 ± 0.3^a^
***S. sobrinus***	5.9 ± 0.3^d^	11.3 ± 0.1^c^	16.0 ± 0.3^a^	12.2 ± 0.3^b^
***Y. enterocolitica***	11.2 ± 0.3^b^	8.1 ± 0.2^c^	14.2 ± 0.4^a^	14.3 ± 0.3^a^
***S. mutans***	7.2 ± 0.3^d^	9.1 ± 0.3^c^	15.4 ± 0.3^a^	10.1 ± 0.2^b^
***P. aeruginosa***	9.6 ± 0.1^b^	8.1 ± 0.2^c^	15.2 ± 0.2^a^	6.0 ± 0.2^d^
***S. aureus***	11.3 ± 0.5^c^	11.1 ± 0.4^c^	16.4 ± 0.1^a^	12.1 ± 0.4^b^
***B. subtilis***	6.1 ± 0.3^c^	10.3 ± 0.3^b^	12.2 ± 0.4^a^	12.5 ± 0.1^a^
***L. monocytogenes***	8.0 ± 0.4^b^	13.3 ± 0.2^a^	14.2 ± 0.4^a^	6.2 ± 0.3^c^
***K. pneumoniae***	14.3 ± 0.3^a^	11.7 ± 0.4^c^	14.2 ± 0.2^a^	13.2 ± 0.2^b^
***S. flexneri***	8.0 ± 0.3^d^	12.0 ± 0.4^c^	16.1 ± 0.3^a^	13.1 ± 0.3^b^

ATCC: American Type Culture Collection, Virginia, USA. PTCC: Persian Type Culture Collection, Tehran, Iran. *Streptococcus sanguinis* PTCC 1449, *Streptococcus salivarius* PTCC 1448, *Streptococcus sobrinus* PTCC 1601, *Yersinia enterocolitica* ATCC 23715, *Streptococcus mutans* PTCC 1683, *Pseudomonas aeruginosa* PTCC 1181, *Staphylococcus aureus* ATCC 25923, *Bacillus subtilis* ATCC 19652, *Listeria monocytogenes* ATCC 13932, *Klebsiella pneumoniae* PTCC 1053, and *Shigella flexneri* PTCC 1234.

*Values are mean ± standard error of triplicates. ^a-d^ Means in the same row with different lowercase letters differed significantly (P < 0.05).

On the other hand, the C14 strain against *Y. enterocolitica*, *P. aeruginosa*, *S. aureus*, *B. subtilis*, *K. pneumonia*, and *S. flexneri*, the M42 strain against *S. salivarius*, *P. aeruginosa*, *S. aureus*, *B. subtilis*, *L. monocytogenes*, and *S. flexneri*, the M22 strain against *S. sanguinis*, *S. salivarius*, *S. sobrinus*, *Y. enterocolitica*, *S. mutans*, *P. aeruginosa*, *S. aureus*, *L. monocytogenes*, and *S. flexneri*, and the Y8 strain against *S. aureus* and *S. flexneri* did not display antagonistic activity after treatment with catalase enzyme. As a result, the inhibitory character of the described strains against these diseases is attributed to hydrogen peroxide generation.

Finally, after treatment with proteinase K enzyme and investigation of anti-pathogenic properties, the inhibitory halos for the C14 strain against *L. monocytogenes*, the M42 strain against *Y. enterocolitica* and *L. monocytogenes*, the M22 strain against *K. pneumoniae*, as well as the Y8 strain against *S. sanguinis*, *S. sobrinus*, *Y. enterocolitica*, *S. mutans*, *P. aeruginosa*, *L. monocytogenes,* and *K. pneumoniae* was not observed, which proved the bacteriocin (proteinaceous) nature of the bacterial extracts against mentioned pathogens (Table 3).

### 3.4 Antibiotic susceptibility

Table 4 shows the susceptibility of LAB isolates to nine therapeutically significant and extensively used antibiotics. All four *Lactobacillus* isolates were positive for resistance to cefixime, ciprofloxacin, and vancomycin. They were, on the other hand, all sensitive to doxycycline and amoxicillin-clavulanic acid. Meanwhile, the germs showed limited resistance to other drugs. The M22 and Y8 strains produced the greatest results, since they were susceptible or semi-responsive to six antibiotics (Table 4).

**Table 4:** Antibiotic susceptibility profiles of isolated *Lactobacillus* strains.

Isolated Strains	Antibiotics								
	CFM	AZM	AMX	D	SXT	CP	CN	AMC	V
**C14**	0.00 ± 0.00^a^	0.00 ± 0.00^c^	22.52 ± 1.18^b^	15.34 ± 1.55^d^	0.00 ± 0.00^c^	15.08 ± 0.74^a^	13.87 ± 1.06^b^	0.00 ± 0.00^c^	0.00 ± 0.00^a^
**M22**	0.00 ± 0.00^a^	28.32 ± 0.24^a^	25.08 ± 0.73^a^	29.67 ± 0.38^b^	38.73 ± 0.55^a^	12.85 ± 1.44^b^	16.29 ± 0.55^a^	26.37 ± 1.28^a^	0.00 ± 0.00^a^
**M42**	0.00 ± 0.00^a^	24.76 ± 0.18^b^	21.13 ± 0.44^c^	34.14 ± 1.47^a^	26.18 ± 1.77^b^	0.00 ± 0.00^c^	0.00 ± 0.00^c^	26.54 ± 0.83^a^	0.00 ± 0.00^a^
**Y8**	0.00 ± 0.00^a^	24.83 ± 0.58^b^	24.96 ± 0.77^a^	26.86 ± 0.34^c^	38.44 ± 0.38^a^	0.00 ± 0.00^c^	14.21 ± 0.66^b^	24.68 ± 0.18^b^	0.00 ± 0.00^a^

CFM, cefixime; AZM, azithromycin; AMX, amoxicillin; D, doxycycline; SXT, trimethoprim sulfamethoxazole; CP, ciprofloxacin; CN, cephalexin; AMC, amoxicillin-clavulanic acid; V, vancomycin. Cefixime results based on R ≤ 15 mm; I: 16–18 mm; S ≥ 19 mm. Azithromycin results based on R ≤ 13 mm; I: 14–17 mm; S ≥ 18 mm. Amoxicillin results based on R ≤ 18 mm; I: 19–21 mm; S ≥ 22 mm. Doxycycline results based on R ≤ 10 mm; I: 11–13 mm; S ≥ 14 mm. Trimethoprim sulfamethoxazole results based on R ≤ 25 mm; I: 26–29 mm; S ≥ 30 mm. Ciprofloxacin results based on R ≤ 15 mm; I: 16–20 mm; S ≥ 21 mm. Cephalexin results based on R ≤ 14 mm; I: 15–17 mm; S ≥ 18 mm. Amoxicillin-clavulanic acid results based on R ≤ 13 mm; I: 14–17 mm; S ≥ 18 mm. Vancomycin results based on R ≤ 14 mm; I: 15–16 mm; S ≥ 17 mm. Performance Standards for Antimicrobial Susceptibility Testing, from Clinical and Laboratory Standards Institute, Twenty-Third Informational Supplement, Wayne, PA (CLSI 2013).

*Values are mean ± standard error of triplicates. ^a-e^ Means in the same row with different lowercase letters differed significantly (p < 0.05).

### 3.5 Determining the probiotic characteristics of the strains

Different cell surface hydrophobicity capabilities were demonstrated by selected isolates. Isolate Y8 had significantly higher hydrophobic rates (62%) than the other tested isolates. The results showed that strains Y8 (49%) and C14 (47%) had the strongest adhesion to human intestinal Caco2 cells. Meanwhile, two bacterial strains, M22 (14%), and M47 (8%), demonstrated poor adherence. Table 5 shows the ability of isolates from culture media to eliminate cholesterol. Isolate Y8 had the highest cholesterol uptake and could absorb > 65% of cholesterol after 20 h of incubation, although isolates M22 and M47 could absorb only 6 and 9% of cholesterol, respectively. The results of hemolytic activity showed that three isolates, including C14, M22, and Y8, had no hemolytic activity (Table 5).

**Table 5:** Surface hydrophobicity (%), biofilm formation, cholesterol uptake (%), hemolytic activity, auto-aggregation (%), and co-aggregation (%) of isolated *Lactobacillus* strains. Values shown are means ± standard deviations (n = 3).

Strains	Hydrophobicity (%)	Biofilm Formation	Cholesterol Removal (%)	Hemolysis		Co-aggregation		
					Auto-aggregation (%)	*E. coli* (PTCC 1276)	*L. monocytogenes* (ATCC 13932)	*B. subtilis* (ATCC 19652)
**C14**	23 ± 0.9^b^	47 ± 1.4^a^	42 ± 1.3^b^	γ	24 ± 1.3^b^	20.1 ± 1.29^b^	18.9 ± 1.34^b^	14.1 ± 1.64^b^
**M22**	11 ± 1.5^c^	14 ± 0.6^b^	06 ± 1.1^d^	γ	19 ± 0.9^c^	22.1 ± 1.5^b^	09.8 ± 0.98^d^	10.1 ± 1.46^c^
**M47**	05 ± 1.1^d^	8 ± 1.2^c^	09 ± 1.3^c^	α	07 ± 1.2^d^	17.2 ± 1.6^c^	11.5 ± 1.52^c^	13.7 ± 1.71^b^
**Y8**	62 ± 2.1^a^	49 ± 0.8^a^	66 ± 2.1^a^	γ	57 ± 2.3^a^	72.1 ± 1.88^a^	69.7 ± 2.41^a^	57.5 ± 1.87^a^

*Values followed by the same letters are not significantly different (*P* > 0.05). Statistical analysis of each formulation was done separately.

The autoaggregation assay results showed that each isolate was capable of autoaggregation. With 57 ± 2.3% autoaggregation, isolate Y8 had the highest autoaggregation percentage of these potential probiotic isolates. Meanwhile, Table 5 shows the coaggregation found in these four isolates. At different times, three different indicator strains were used to assess coaggregation. The ability to aggregate with pathogenic bacteria was demonstrated by all tested isolates, but the coaggregation ranges were found to be time-dependent and strain-specific. In comparison to the other isolates tested, Y8 had the highest percentages of coaggregation with *E. coli* (72%), L. monocytogenes (69%), and *B. subtilis* (57%).

### 3.6 Pre-screening cytotoxic test of selected *Lactobacillus* strains on KB cells

**Fig. 1:**
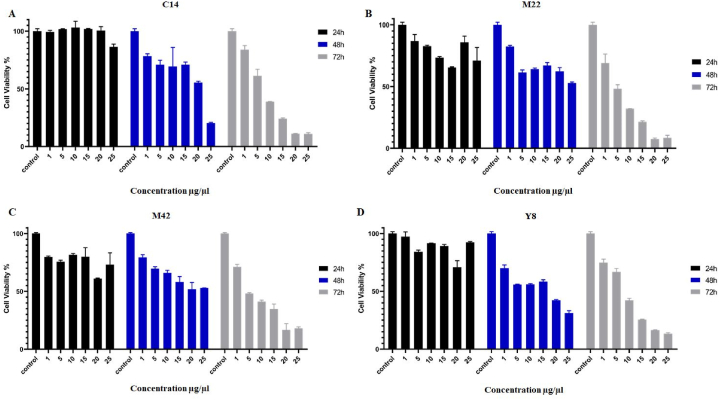
The cytotoxic effects of C14 (Panel A), M22 (Panel B), M42 (Panel C), and Y8 (Panel D) secretions (1–25 μg/mL) on KB cancer cell line after 24h, 48h, and 72 h incubation. Each bar represents the mean ± SE of six replicates. Control: untreated cancer cell line.

The pre-screening test was performed in different concentrations (0, 1, 5, 10, 15, 20, and 25 μg/mL) after 24, 48, and 72 h of incubation to assess the most effective Lactobacillus secretions, extracted concentration, and incubation time. The lethal effects of the isolated metabolites in treated KB cancer cell lines were dosage- and time dependent, as shown in Fig. 1 and Table 6. The isolated metabolites of the Lactobacillus strain Y8 produced the lowest cell viability (%) of the four strains. According to the findings, the extracted metabolite of the Y8 strain had considerably decreased cell viability (%) (0.05 level) at the concentration of 25 μg/mL when compared to other concentrations (0, 1, 5, 10, 15, and 20 μg/mL), hence 25 μg/mL was selected as the effective concentration. After 48 and 72 h of incubation, the percentage of living cells was the lowest and the anti-cancer activity was the highest.

**Table 6:** The IC_50_ values of C14, M22, M42, and Y8 secretions (1–25 μg/mL) on KB cancer cell line after 24h, 48h, and 72 h incubation.

Extractions	KB		
	24 h	48 h	72 h
**C14**	ND	21.79 μg/μL	6.37 μg/μL
**M22**	ND	ND	3.44 μg/μL
**M42**	ND	ND	4.55 μg/μL
**Y8**	ND	16.44 μg/μL	6.4 μg/μL

*ND: not detected value.

### 3.7 Cytotoxic effects of Y8 strain on different cancer and normal cell lines

**Fig. 2:**
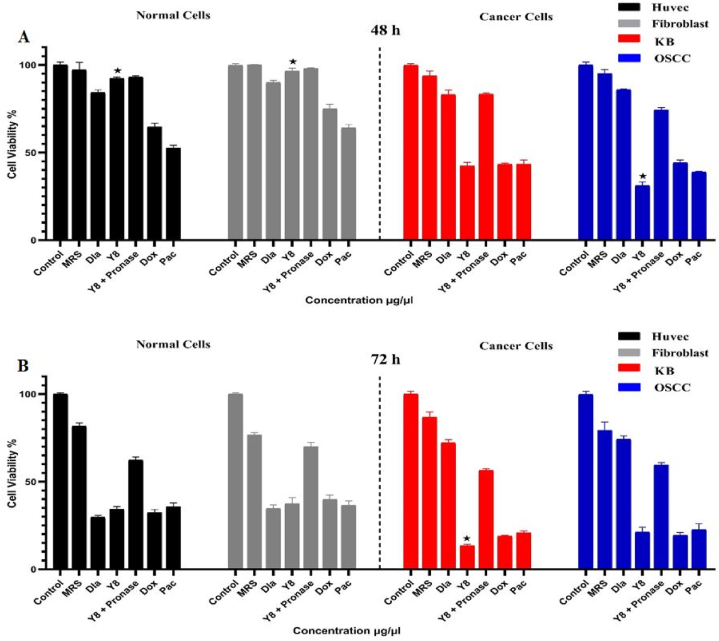
The cytotoxic effects of Y8 and pronase treated Y8 secretion (25 μg/mL) on oral cancer (KB and OSCC) and normal (fibroblast and HUVEK) cell lines after 48h (Panel A) and 72 h (Panel B) incubation. Each bar represents the mean ± SE of six replicates. Asterisks denote statistically significant differences at P < 0.05 level as compared with positive controls (doxorubicin and paclitaxel). Control: untreated cancer cell line. MRS: MRS treated cell lines. Dla: treated cells with in-market probiotic strain (*Lactobacillus acidophilus* PTCC 1643). Y8 + Pronase: pronase treated Y8 secretion. Dox: treated cells with doxorubicin anticancer drug. Pac: treated cells with paclitaxel anticancer drug.

The MTT assay was used to compare MRS-treated, untreated, and drug-treated (doxorubicin and paclitaxel) cell lines as negative and positive controls to 25 μg/mL extracted metabolites of the Y8 strain after 48 and 72 h incubation on oral cancer (KB and OSCC) and normal (fibroblast and HUVEK) cells. The mean changes in cancer cell viability for Y8 and anti-cancer medications (doxorubicin and paclitaxel) after 48 and 72 h of incubation in comparison to negative controls were substantially low (less than 46%) at the 0.05 level, demonstrating their strong anti-cancer activity. Meanwhile, the cell viability of Y8 on OSCC cells after 48 h of incubation and on KB cells after 72 h of incubation was considerably reduced at 0.05 levels and demonstrated superior anti-cancer actions than doxorubicin and paclitaxel (Fig. 2).

The mean differences for normal cells (fibroblast and HUVEK) treated with Y8 compared to doxorubicin and paclitaxel-treated cells after 48 h of incubation, on the other hand, were substantially greater at 0.05 levels and showed no adverse effects on normal cell lines. Y8, doxorubicin, and paclitaxel, on the other hand, dramatically inhibited rapidly dividing normal cell lines after 72 h of incubation (Fig. 2). OSCC cells were most vulnerable to isolate Y8. The findings also revealed that cytotoxicity was lower in normal cells than in cancer cells after 48 h of incubation, indicating the specificity of toxicity effects at this incubation period for cancer cells (Fig. 2).

After 48 and 72 h, the mean differences for pronase-treated Y8 metabolites against Y8 normal extracted metabolites and anti-cancer medications (doxorubicin and paclitaxel) were considerably greater in KB and OSCC cancer cell lines. It was discovered that effective proteins played an important part in the cytotoxic effects of Y8 secretions. In normal fibroblast and HUVEK cell lines, the mean differences for pronase-treated Y8 metabolite over untreated Y8 metabolite were not significant after 48 h of incubation but were considerably greater (60% < X < 72%) after 72 h. This demonstrated the presence of additional cytotoxic mechanisms on normal cell lines after 48 h of incubation, although effective proteins were the dominant cytotoxic factor on normal cell lines after 72 h of incubation (Fig. 2).

### 3.8 Cell morphological analysis by AO/EB staining

The viable and apoptotic cells were identified using AO/EB fluorescent staining. Green, intact cells were identified as viable cells, while orange, shrinking cells were identified as apoptotic cells. Three repeats of this test were also carried out using three-time fluorescent imaging.

**Fig. 3:**
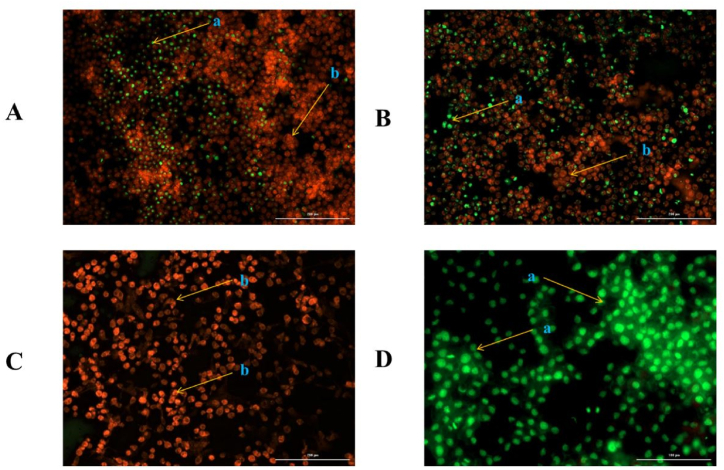
Dual acridine orange/ethidium bromide (AO/EB) fluorescent staining after incubation of Y8 secreted metabolite (25 μg/mL) on KB cancer cell line. Panels represent; A) treated KB cancer cell line after 24-h incubation, B) treated KB cancer cell line after 48-h incubation, C) treated KB cancer cell line after 72-h incubation, D) Untreated KB cancer cell line as control, a = apoptotic cells, b = normal intact cells.

When compared to viable, necrotic, and spontaneous cell death in untreated cell lines, the mean number of apoptotic cells in KB cancer cell lines treated with Y8 secreted metabolite (25 g/mL) was significantly higher at 0.05 levels (Fig. 3, Panels A–D). Meanwhile, after 72 h of incubation, the apoptotic cells were significantly higher (Fig. 3C).

According to the results in Fig. 3, it was obvious that Y8 secretion significantly reduced viable cells. Furthermore, certain cells displayed apoptotic cell characteristics such as condensed chromatins, cell shrinkage, membrane blebbing, and the development of apoptotic bodies. Despite this, the quantity of red blood cells did not increase. This demonstrates that the majority of the deceased cells were caused by apoptosis rather than necrosis.

### 3.9 Molecular identification

Using 16S rRNA gene sequencing, the phenotypic characterization of the chosen *Lactobacillus* strains was confirmed. Amplification of the 16S rRNA genes indicated that the four isolates were all Lactobacillus. Isolates C14 and M22 were identified as *Limosilactobacillus fermentum*, isolate M42 as *Lactiplantibacillus pentosus*, and isolate Y8 as *Lactiplantibacillus plantarum*. They were assigned the accession numbers OP811008, OP811009, OP811010, and OP811011 to the NCBI GeneBank, respectively.

## 4 Discussion

Several studies have shown that *Lactobacillus* strains have health benefits and can help prevent cancer. In this study, the anticancer activities of 21 *Lactobacillus* isolates were determined using in vitro techniques. We discovered that isolate Y8 can function as a probiotic and has anti-cancer effects.

To determine the safety and efficacy of these isolates, they must be thoroughly identified and described. Various in vitro tests, including assessment using a dynamic gastrointestinal model under gastric and intestinal growth conditions [18,19], as well as exposure to adjusted PBS with high bile salt and low pH [20], have been used to select isolates resistant to harsh conditions.

The pH of the human stomach ranges from 1.5 when fasting to 4.5 after a meal; food digestion may take up to 3 h [21]. Although the pH of the stomach may drop as low as 1.0, pH 3.0 is often used by researchers for in vitro experiments [22]. This study used a pH of 3.0 to look into potential probiotics through the extraction of acid-tolerant microbes [23]. According to earlier investigations, *Lactobacillus* species of human and animal origin retain viability despite being exposed to pH 3.0 [[24], [25], [26]]. These findings are supported by our findings. The most significant impediment to Lactobacillus survival in the host's GI tract has been identified as the acid environment of the stomach and the bile salt in the duodenum (digestive conditions) [27]. Tolerance to acidic, bile-salty, and enzymatic conditions is therefore essential for Lactobacillus species to grow, thrive, and be active in gastrointestinal transit. We discovered that four isolates, C14, M22, M42, and Y8, performed well, with >71% viability in high concentrations of bile salt and low pH solutions and >32% viability in digestive conditions.

Many studies indicate that specific probiotic strains can provide the capability of anti-infection against intestinal microbes [[28], [29], [30]]. The antibacterial activity against pathogenic bacteria has influenced comprehensive consideration [31]. These strains, as accepted probiotics, should have antimicrobial activities against both gram-positive and gram-negative pathogenic microorganisms. The most potent antimicrobial action and co-aggregation capacity were found in Y8. Also, antimicrobial activities are associated with co-aggregation. The close contact between pathogenic bacteria and probiotics occurs during the release of anti-microbial substances, and high co-aggregation of the probiotics can suppress pathogens [32].

Probiotics are designed to include intrinsic and mobile genetic factors that enable them to promote antibiotic resistance. Excessive antibiotic use by patients has resulted in the appearance of antibiotic resistance genes in the intestinal flora, and by transferring these genes to other microorganisms living in the digestive tract, particularly pathogens; acute antibiotic resistance problems in society are created. As a result, antibiotic susceptibility is regarded as one of the most important factors to consider when selecting probiotics [33]. The high antibiotic susceptibility of the M22 and Y8 isolated strains is most likely due to the limited use of animal antibiotics in Kermanshah Province, where the samples (milk and yogurt) were collected. Other researchers [34,35] have reported high antibiotic resistance in LAB bacteria isolated from traditional dairy products, which contradicts these findings. The susceptibility of Lactobacillus strains to various antibiotics, as demonstrated by our findings, is one of the most important factors in determining safety [36]. However, various probiotic strains in the LAB group, such as the *Lactobacillus* genus, primarily carry vancomycin resistance genes, confirming our findings [37]. Klose et al. demonstrated the selective isolation of intrinsically vancomycin-resistant Lactobacillus species from badger intestine [38].

The ability of bacteria to adhere to the intestinal mucosa is determined by examining the adhesion capacity of probiotics to the hydrophobic phase of the used solvent (surface hydrophobicity), which prevents pathogens from sticking to the intestine and contaminating the digestive system [39]. Similar to the results of the Y8 strain, previous studies have shown that probiotic bacteria mainly exhibit acceptant cell surface hydrophobicity, and these studies have also proven the relationship between cell surface hydrophobicity and bacterial adhesion ability. As in our results, the difference in the production of surface proteins is the main factor in creating a wide range of cell surface hydrophobicity in *Lactobacillus* strains [40].

Another distinguishing feature of probiotics is their ability to bind to intestinal epithelial cells. As a result, adherence ability is regarded as an important criterion for selecting potential probiotic bacteria. Probiotics must adhere to the intestinal mucosa and not be easily removed from the gut by smoky bowel movements in order to colonize in the gut. Our findings are consistent with previous research, which found that only a few *Lactobacillus* strains, such as Y8 and C14, could adhere well to Caco-2 cells [41].

Another important and necessary feature for the introduction and selection of probiotics is their high ability to uptake cholesterol, similar to the Y8 and C14 strains. Evidence suggests that probiotics' hypocholesterolemic effects may be due to cholesterol absorption or binding to the surface of bacterial cells. The conversion of cholesterol to coprostanol by the reductase enzyme, incorporation of cholesterol in the cell wall, and disruption of cholesterol micelle formation in the intestine by de-conjugated bile salts are the most important mechanisms of cholesterol reduction by probiotics [42].

Three of the four tested strains (C14, M22, and Y8) lacked α- or β-hemolytic properties and only demonstrated γ-hemolytic activity. Other studies have found that most *Lactobacillus* strains, like ours, have no hemolytic activity. However, the absence of blood cell hemolysis does not prove the safety of probiotic strains, according to the guidelines.

Probiotic co-aggregation and auto-aggregation are critical in preventing pathogens from colonizing the surface [43]. Auto-aggregation allows microorganisms of the same species to form self-forming groups, and this situation is frequently associated with microorganisms adhering to the intestinal mucosa [44]. Co-aggregation is the intercellular adhesion of different strains that is also capable of interacting with pathogens. As a result, aggregation may represent one type of anti-infection defense mechanism in a host [45]. In this study, four *Lactobacillus* strains were found to have varying levels of co-aggregation and auto-aggregation, with the highest observed in the Y8 strain (>57%). The complicated interactions of bacteria's surface molecules, such as secreted factors and proteins, can cause this. An earlier study established that any aggregation phenotype ability is possibly related to environmental and internal factors [46].

The research on anticancer properties is a good example of the much broader health-promoting properties attributed to probiotic Lactobacilli than previously suggested [47,48].Some probiotic LAB strains inhibit cancer cell line growth, including liver, breast, gastric, bladder, and colon cancer. We used the MTT test to show that isolate Y8 was cytotoxic to oral cancer (KB and OSCC) and normal (fibroblast and HUVEK) cell lines. This approach is based on cells' capacity to convert yellow tetrazolium to blue formazan [49].

According to the findings, Y8 has potent anti-cancer properties in KB and OSCC cell lines. Extracted metabolites from this strain demonstrated time- and dose-dependent effects, with 25 g/mL dry weight after 48 h incubation producing better results than other dosages and times. These are just the primary experiments showing the dose and time dependent cytotoxic effects of Y8 strain on oral cancer cells and further detailed study is required to finally conclude the statement. Y8-secreted metabolites are qualified as safe and inexpensive anti-cancer drugs since they showed no adverse effects on normal cell lines despite the substantial cytotoxicity associated with other anti-cancer treatments on sensitive cell lines and tissues [50,51].

The particular processes through which probiotic metabolites inhibit and prevent cancer cell lines are unknown. They use effective proteins to reduce harmful and carcinogenic fecal enzymes such as -glycosidase, -glucuronidase, IO hydratase-dehydrogenase, nitroreductase, nitrate/nitrite reductase, and azoreductase [52]. These findings support our findings, which show that effective proteins played a key role in the cytotoxic effects of an isolated *Lactobacillus* strain.

The type of cell that dies reveals the nature of the feedback from neighboring tissue. Necrosis causes oxidative stress and the release of numerous pro-inflammatory cytokines [53]. Apoptosis, on the other hand, is a controlled process that is commonly aided by the lowest loss of membrane integrity prior to secondary necrosis or a later stage. Cell death of this type is frequently associated with phagocytosis by resident tissue macrophages and the release of anti-inflammatory cytokines [54]. Under a fluorescent microscope, AO/EB can be used to distinguish apoptosis-related changes in cell membranes within apoptosis [55]. Our findings corroborate the notion that the cytotoxicity of isolate Y8 is only limited, causing apoptosis.

In conclusion, isolate Y8 exhibited the highest probiotic score among the investigated Lactobacillus strains, including anti-pathogenic activity, cholesterol assimilation, resistance to high bile salt and low pH, hydrophobicity, antibiotic susceptibility, and auto- and co-aggregation. This safe isolate's isolated bacteriocin (protein nature) may also effectively limit the development of KB and OSCC cancer cell lines. Because of this, it may be a good choice for treating oral cancers.

## Author contribution statement

Babak Haghshenas, Amir Kiani: Conceived and designed the experiments.

Nesa Moazami: Performed the experiments.

Yousef Nami:Contributed reagents, materials, analysis tools or data.

Omid Tavallaei: Wrote the paper, Analyzed and interpreted the data.

## Data availability statement

The datasets generated and/or analyzed during the current study are available in the NCBI GeneBank repository, accession numbers OP811008, OP811009, OP811010, and OP811011.

## Ethics statement

This study was reviewed and approved by Dr. Mahmood Reza Moradi (Chairman of the Academic/Regional Ethics Committee in Biomedical Research) and Dr. Reza Khodarahmi (Secretary of the Academic/Regional Ethics Committee in Biomedical Research) of Kermanshah University of Medical Sciences (Approval ID: IR.KUMS.REC.1400.106). All methods were performed in accordance with the relevant guidelines and regulations by including a statement in the methods section.

## Funding

This work was supported by the Deputy for Research and Technology of 10.13039/501100005317Kermanshah University of Medical Sciences (Grant No. 4000328).

## Declaration of competing interest

The authors declare that they have no known competing financial interests or personal relationships that could have appeared to influence the work reported in this paper.
